# Relationship Between CHA2DS2-VASc Score and Right Ventricular Dysfunction in Patients With Acute Pulmonary Thromboembolism

**DOI:** 10.1177/1076029618785771

**Published:** 2018-07-11

**Authors:** Murat Gök, Alparslan Kurtul, Murat Harman, Meryem Kara, Muhammed Süleymanoglu, Ender Ornek

**Affiliations:** 1 Cardiology Department, Ankara Numune Education and Research Hospital, Ankara, Turkey; 2 Cardiology Department, Ankara Education and Research Hospital, Ankara, Turkey; 3 Cardiology Department, Fırat University Medical Faculty, Elazığ, Ankara, Turkey; 4 Cardiology Department, Yüksek İhtisas Training and Research Hospital, Ankara, Turkey

**Keywords:** CHA2DS2-VASc score, pulmonary thromboembolism, right ventricular dysfunction

## Abstract

In this study, the association between the right ventricular dysfunction (RVD) and CHA2DS2-VASc (C: congestive heart failure or left ventricular systolic dysfunction, H: hypertension, A: age of ≥ 75 years, D: diabetes mellitus, S: previous stroke, V: vascular disease, A: age between 65 and 74 years, Sc: female gender) scores was investigated in patients with acute pulmonary thromboembolism (PTE). The patients have been assigned to 3 subgroups as massive, submassive, and nonmassive PTE. The CHA2DS2-VASc scores were calculated for all of the patients, and the scores have been classified into 3 groups as the scores between 0 and 1, the scores of 2, and the scores of 3 and over. The independent predictors of the RVD were investigated by the univariate and multivariate regression analyses. The independent predictors of the RVD were determined to be the CHA2DS2-VASc scores (*P* = .034), the systolic pulmonary artery pressure (*P* < .001), the presence of acute deep vein thrombosis (*P* = .007), high simplified Pulmonary Embolism Severity Index (*P* < .001), D-dimer (*P* < .006), and the mean platelet volume (*P* < .001). The CHA2DS2-VASc scores predicted the RVD with 70% sensitivity and 50% specificity as determined by the receiver operating characteristic analysis. The CHA2DS2-VASc score is an independent predictor of the RVD in patients with acute PTE.

## Introduction

Acute pulmonary thromboembolism (PTE), which ranks the third among the cardiovascular system–related deaths in the United States, has an incidence of 23 in 100 000, and approximately 100 000 to 200 000 PTE cases end up with death annually.^1^ The clinical presentation of the PTE is closely associated with the number of clogged pulmonary arteries and the size of the thrombus as well as with the cardiopulmonary reserve.^2,3^ Pulmonary thromboembolism is a clinical phenomenon presenting with a spectrum of findings, ranging from small emboli causing mild hemodynamic dysfunction to massive emboli leading to cardiogenic shock, and it can sometimes be fatal.^4^ The clinical presentations of PTE are classified as massive, submassive, and nonmassive.^4,5^ An acute right ventricular dysfunction (RVD), with accompanying hypotension, shock, or cardiovascular arrest, is present in massive PTE. In submassive PTE, in contrast to the presence of systemic blood pressure, signs of RVD (dilatation and hypokinesia) are present as confirmed by echocardiography. In nonmassive PTE, the systemic blood pressure and the right ventricular functions are normal. The classification of the cases with PTE as such is very important as it is decisive of the treatment management. While the thrombolytic therapy is at the forefront of the treatment in massive PTE, anticoagulants are the treatment of choice in the other clinical presentations. Echocardiography is a confirmatory tool in PTE in determining whether the cardiac functions are affected. Echocardiography is a cardiologist-dependent test, and the right ventricular functions of patients cannot be assessed at hospitals, where there are no cardiologists or where echocardiograms cannot be performed. Especially in submassive PTE, the predictor of mortality is associated with whether RVD accompanies the clinical presentation or not,^6^ and these patients should be given thrombolytic treatments.

The CHA2DS2-VASc (C: congestive heart failure or left ventricular systolic dysfunction, H: hypertension, A: age of ≥ 75 years, D: diabetes mellitus, S: previous stroke, V: vascular disease, A: age between 65 and 74 years, Sc: female gender) score is used to determine the thromboembolism risk and to manage the anticoagulant treatment in patients diagnosed with nonvalvular atrial fibrillation.^7^ In some studies, it was demonstrated that it could be used as a determinant of mortality and morbidity in patients with congestive heart failure, who were applied cardiac resynchronization therapy. In another study, it was reported that it could be used in the thrombotic cases, developing after percutaneous coronary interventions.^8,9^ Recent studies have demonstrated that the CHA2DS2-VASc score can be recognized as a prognostic indicator of the in-hospital and long-term mortality in patients with acute coronary syndrome.^10–12^ In addition, it has been demonstrated that the CHA2DS2-VASc score predicts the development of contrast-induced nephropathy in patients with acute coronary syndrome and in patients undergoing percutaneous coronary angioplasty.^13,14^ In this study, we investigated the association of the CHA2DS2-VASc scores with the clinical subgroups of PTE, RVD, and in-hospital mortality in patients with the PTE.

## Methods

### Study Population and Design

The study was conducted with patients presenting to our hospital during the period between January 2015 and June 2017. The patients over 18 years of age, who presented to the emergency department with symptoms of shortness of breath, hemoptysis, and chest pain and who were diagnosed with PTE after undergoing pulmonary computed tomography angiography with a prediagnosis of PTE, were included in the study.

**Figure 1.**
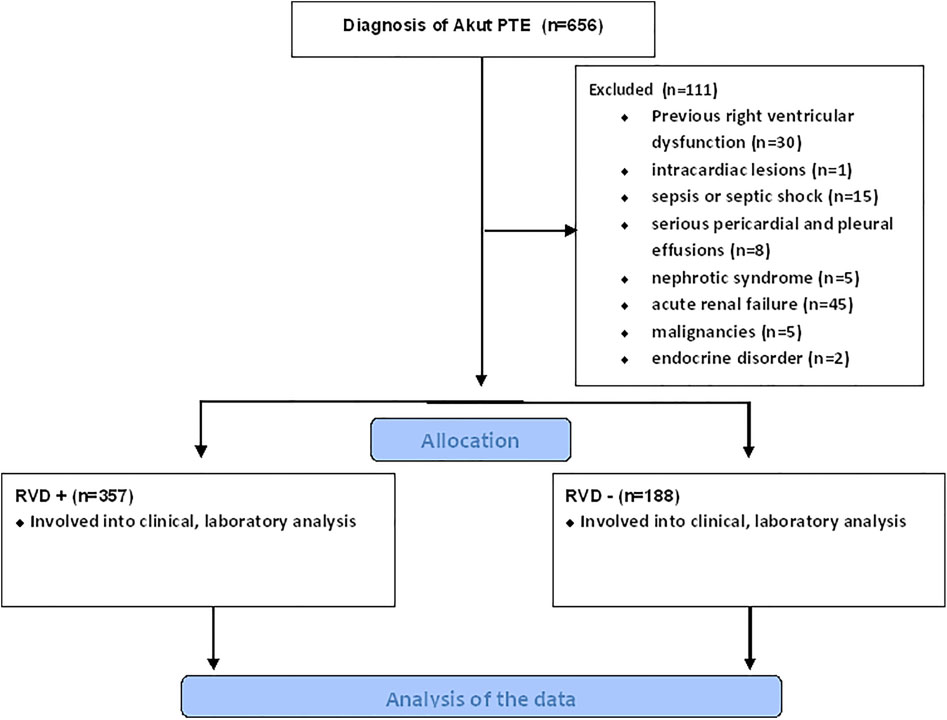
Participant selection process to the present study.

The patients were excluded if they were previously diagnosed with RVD (n = 30) or if they had intracardiac lesions (n = 1), sepsis or septic shock (n = 15), serious pericardial and pleural effusions (n = 8), nephrotic syndrome (n = 5), acute renal failure (n = 45), malignancies (n = 75), and any endocrine disorder affecting the hemodynamics of the patient (n = 2; Figure 1).

The demographic characteristics of the patients, comorbidities, patient histories, vital signs, treatment regimens at the hospital, monitoring findings daily, and the duration of hospital stays were obtained from the medical files of the patients. The tests of hemogram, biochemistry, cardiac enzymes, and hemostasis were performed. All patients had their echocardiograms (using the General Electric Vivid 7 ultrasound system, Chicago, IL ) performed in order to evaluate the RVD and the pulmonary arterial pressure. The pulmonary artery systolic pressure (PASP) was calculated by the continuous-wave Doppler method. The tricuspid annular plane systolic excursion (TAPSE) method was used to evaluate the RVD. A value of TAPSE ≤15 was accepted to confirm the presence of the RVD.

The acute PTE cases were assigned to 3 subgroups, according to their hemodynamic and radiological characteristics. The patients developing hypotension were assigned to the massive PTE subgroup, the patients with stable hemodynamics but with RVD detected by echocardiogram were assigned to the submassive PTE group, and the patients with stable hemodynamics with no RVD confirmed by echocardiogram were assigned to the nonmassive PTE group.^5^ The simplified Pulmonary Embolism Severity Index (sPESI) scores of the patients were calculated using data obtained from FONET software program (version 3.1.1.6 b5). The patients with sPESI risk scores of 0 were accepted to have low sPESI scores. The patients with sPESI scores of 1 were accepted to have high sPESI scores.

The CHA2DS2-VASc scores of the patients were calculated using the patient data recorded in the FONET system. The components of the CHA2DS2-VASc score were calculated as follows: congestive heart failure (1 point), hypertension (1 point), age (>75 years [2 points]), diabetes mellitus (1 point), history of stroke or transient ischemic attacks (2 points), history of vascular disease (1 point), age (>65 years [1 point]), and female gender (1 point).

### Statistical Analysis

Quantitative variables were expressed as mean value (standard deviation) for parametric variables and median and interquartile ranges for nonparametric variables. Continuous variables were analyzed for normal distribution using the Kolmogorov-Smirnov test and analyzed for homogeneity using the Levine tests. Comparisons of parametric values among groups were performed by one-way ANOVA. Comparisons of nonparametric values among groups were performed by the Kruskal-Wallis test. Tukey HSD (for parametric variables) and Bonferroni adjustment Mann-Whitney *U* test (for nonparametric variables) were used as post hoc test for multiple comparisons among the groups. A 2-tailed *P* < .05 was considered significant. Univariate logistic regression was used to identify independent predictors of RVD. After performing univariate analysis, significantly obtained variables were used in multivariate logistic regression analysis. Also, correlation analyses were performed between CHA2DS2-VASc/sPESI and CHA2DS2-VASc/troponin levels. The receiver operating characteristic (ROC) curve analysis was performed in order to determine the best cutoff value and area under the curve (AUC) of CHA2DS2-VASc, and the sensitivity and specificity at that point were obtained for predicting the presence of RVD. All analyses were performed using SPSS for Windows 18.0 (version 18.0; SPSS, Chicago, Illinois).

## Results

A total of 545 patients with acute PTE were included in the study, and the patients were assigned to 3 groups, namely massive PTE (n = 172), submassive PTE (n = 238), and nonmassive PTE (n = 135). Of all the patients, 251 patients were males. The associations of the variables with the PTE subgroups are summarized in Table 1. Cardiac diseases were detected at a higher rate in the massive PTE group, and this finding was statistically significant (*P* = .001). Compared to the other groups, the incidence of the RVD was detected to be significantly higher in the massive PTE group (*P* < .001). The presence of acute deep venous thrombosis (DVT). at presentation was statistically significantly higher in the massive PTE group, too (*P* = .001). Again, compared to other groups, the incidence of thrombolytic treatments (*P* < .001) and the in-hospital mortality rate were detected to be higher in the massive PTE group (*P* < .001).

**Table 1.** The Association of Variables With Acute Pulmonary Embolism Subgroups.

Variable	Acute Pulmonary Embolism			*P* Value
	Massive (n)	Submassive (n)	Nonmassive (n)	
CHA2DS2-VASc score				.002
0-1	53 (30.8)		67 (49.6)	
2	38 (22.1)	43 (18.1)	28 (20.7)	
3-7	81 (47.1)	114 (47.9)	40 (29.6)	
Male gender, n (%)	79 (45.9)	114 (47.9)	58 (43)	.655
Dyspnea, n (%)	164 (95.3)	202 (84.9)	104 (77)	<.001
Diabetes mellitus, n (%)	20 (11.6)	20 (8.4)	20 (14.8)	.156
Current smoker, n (%)	67 (39)	80 (33.6)	40 (29.6)	.222
Hemoptysis, n (%)	17 (9.9)	33 (13.9)	14 (10.4)	.396
Retrosternal pain, n (%)	83 (48.3)	134 (56.3)	86 (63.7)	.025
Cardiac disease, n (%)	87 (50.6)	104 (43.7)	40 (29.6)	.001
Liver disease, n (%)	17 (9.9)	25 (10.5)	4 (3)	.030
Neurologic disease, n (%)	29 (16.9)	39 (16.4)	17 (12.6)	.536
Prior deep venous thrombosis, n (%)	7 (4.1)	16 (6.7)	8 (5.9)	.515
Prior pulmonary thromboembolism, n (%)	6 (3.5)	10 (4.2)	8 (5.9)	.575
Right ventricular dysfunction, n (%)	160 (93)	154 (64.7)	43 (31.9)	<.001
Deep venous thrombosis, n (%)	104 (60.5)	138 (58)	55 (40.7)	.001
Thrombolytic therapy, n (%)	136 (79.1)	66 (27.7)	2 (1.5)	<.001
Enoxaparin therapy, n (%)	152 (88.4)	236 (99.2)	135 (100)	<.001
Warfarin therapy, n (%)	120 (69.8)	170 (71.4)	121 (89.6)	<.001
In-hospital mortality, n (%)	16 (9.3)	5 (2.1)	1 (0.7)	<.001

**Figure 2.**
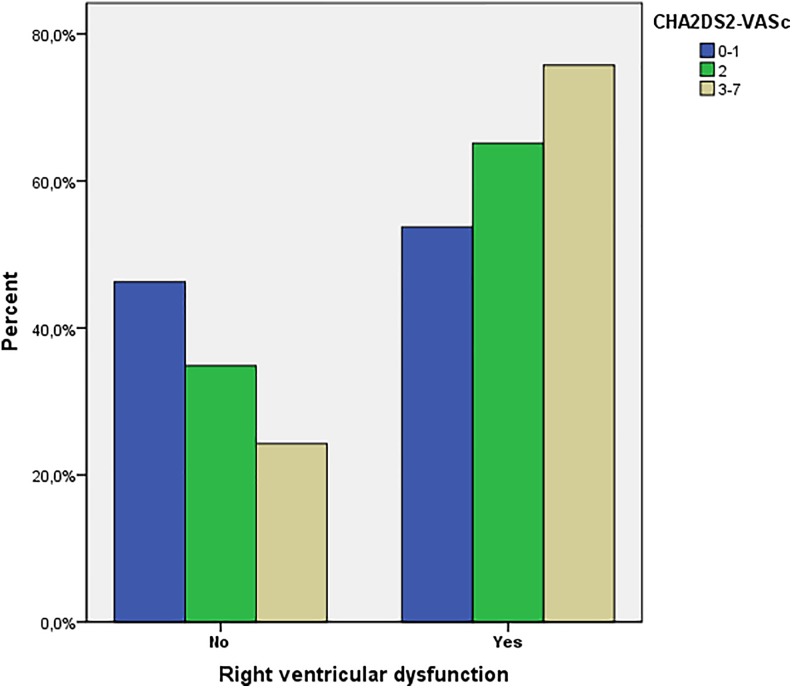
The association of CHA2DS2-VASc score with the presence and absence of right ventricular dysfunction.

The CHA2DS2-VASc scores were classified into 3 groups as follows: the scores between 0 and 1 were classified as the low-risk group, the scores of 2 were classified as the moderate risk group, and the scores of 3 and over were classified as the high-risk group. The associations of the variables with these groups are summarized in Table 2. The CHA2DS2-VASc score was more significant in the submassive PTE group (*P* = .002). Male gender was common with a higher rate in the low-risk group (*P* < .001). The percentage of the diabetic patients was higher in the high-risk group (*P* < .001), and the percentage of smokers was higher in the low-risk group (*P* < .001). The patients with histories of cardiac diseases, liver diseases, and neurological diseases were more common in the high-risk group (*P* < .001, *P* = .007, *P* < .001). The incidence of the RVD was more common in the high-risk group compared to the other groups, and this finding was statistically significant (*P* < .001; Figure 2). The in-hospital mortality rates were not found to be associated with any of the risk groups (*P* = .517).

**Table 2.** The Association Between CHA2DS2-VASc Score and Various Variables.

Variable	CHA2DS2-VASc Score			*P* Value
	0-1 (n = 201)	2 (n = 109)	3-7 (n = 235)	
Type of pulmonary thromboembolism				.002
Massive	53 (26.4)	38 (34.9)	81 (34.5)	
Submassive	81 (40.3)	43 (39.4)	114 (48.5)	
Nonmassive	67 (33.3)	28 (25.7)	40 (17)	
Male gender, n (%)	123 (61.2)	56 (51.4)	72 (30.6)	<.001
Dyspnea, n (%)	169 (84.1)	97 (89)	204 (86.8)	.461
Diabetes mellitus, n (%)	6 (3)	6 (5.5)	48 (20.4)	<.001
Current smoker, n (%)	98 (48.8)	44 (40.4)	45 (19.1)	<.001
Hemoptysis, n (%)	40 (19.9)	5 (4.6)	19 (8.1)	<.001
Retrosternal pain, n (%)	135 (67.2)	55 (50.5)	113 (48.1)	<.001
Cardiac disease, n (%)	24 (11.9)	37 (33.9)	170 (72.3)	<.001
Liver disease, n (%)	8 (4)	9 (8.3)	29 (12.3)	.007
Neurologic disease, n (%)	0 (0)	9 (8.3)	77 (32.3)	<.001
Prior deep venous thrombosis, n (%)	12 (6)	9 (8.3)	10 (4.3)	.321
Prior pulmonary thromboembolism, n (%)	12 (6)	2 (1.8)	10 (4.3)	.235
Right ventricular dysfunction, n (%)	108 (53.7)	71 (65.1)	178 (75.7)	<.001
Deep venous thrombosis, n (%)	114 (56.7)	55 (50.5)	128 (54.5)	.572
Thrombolytic therapy, n (%)	84 (41.8)	38 (34.9)	82 (34.9)	.236
Enoxaparin therapy, n (%)	197 (98)	105 (96.3)	221 (94)	.108
Warfarin therapy, n (%)	168 (83.6)	89 (81.7)	154 (65.5)	<.001
In-hospital mortality, n (%)	7 (3.5)	3 (2.8)	12 (5.1)	.517

**Figure 3.**
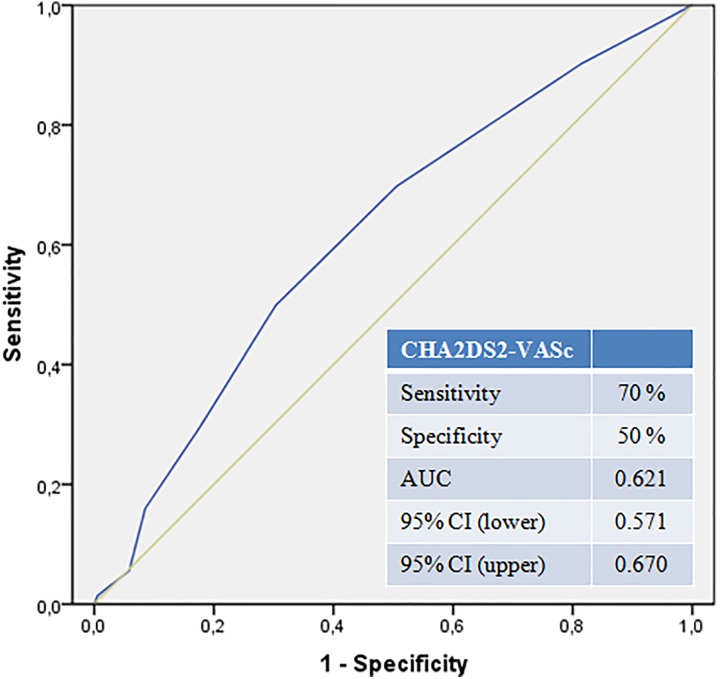
The CHA2DS2-VASc score in the receiver operating characteristics (ROC) curve to predict the right ventricular dysfunction.

Then, we investigated the predictors of the RVD using univariate and multivariate analyses (Table 3). The following factors were the independent predictors of the RVD:PASP (odds ratio [OR]: 1.162, *P* < .001), acute DVT (OR: 3.762, *P* = .007), the CHA2DS2-VASc score (OR: 1.497, *P* = .034), sPESI (OR: 26.279, *P* < .001), mean platelet volume (OR: 1.535, *P* < .001), and D-dimer (OR: 1.000, *P* < .006). It was demonstrated by the ROC analysis that the CHA2DS2-VASc score predicted the presence of RVD with a 70% sensitivity and 50% specificity (AUC: 0.621 [0.671-0.670]; Figure 3). The results obtained from the correlation analysis showed that there were weak but significant correlations between the variables (CHADSVASC score/sPESI and CHADSVASC score/troponin levels, *r* = 0.096, *P* = .025 and *r* = 0.171, *P* = .001, respectively).

**Table 3.** Predictors of Right Ventricular Dysfunction in Patients With Acute Pulmonary Embolism.

Variable	Univariate Analysis		Multivariate Analysis	
	Odds Ratio (95% CI)	*P* Value	Odds Ratio (95% CI)	*P* Value
Current smoker	1.018 (0.702-1.476)	.925		
Dyspnea	2.652 (1.615-4.347)	<.001	1.483 (0.528-4.160)	.455
Hemoptysis	1.939 (1.146-3.280)	.014	3.538 (0.530-23.618)	.192
Retrosternal pain	1.465 (1.022-2.099)	.038	1.179 (0.506-2.747)	.703
Systolic blood pressure	0.980 (0.943-1.019)	.319		
Cardiac disease	2.440 (1.672-3.561)	<.001	2.311 (0.793-6.733)	.125
Liver disease	6.133 (2.164-17.380)	.001	3.485 (0.297-40.849)	.320
Neurologic disease	1.408 (0.846-2.341)	.188		
Prior DVT	1.399 (0.670-2.922)	.371		
Prior PTE	1.376 (0.599-3.161)	.451		
Wells	1.252 (1.154-1.358)	<.001	1.033 (0.843-1.266)	.752
Systolic PAP	1.164 (1.133-1.196)	<.001	1.162 (1.108-1.219)	<.001
C-reactive protein	0.999 (0.996-1.002)	.557		
DVT	2.564 (1.782-3.690)	<.001	3.762 (1.439-9.838)	.007
White blood cell count	1.000 (1.000-1.000)	<.001	1.000 (1.000-1.000)	.460
Po_2_	0.953 (0.935-0.972)	<.001	0.992 (0.948-1.039)	.738
CHA2DS2-VASc score	1.276 (1.141-1.427)	<.001	1.497 (1.032-2.173)	.034
NLR	1.191 (1.111-1.277)	<.001	1.074 (.0854-1.350)	.542
sPESI	12.048 (6.493-22.222)	<.001	26.279 (5.235-131.912)	<.001
PLR	1.007 (1.004-1.011)	<.001	1.013 (1.000-1.027)	.050
MPV	1.131 (1.033-1.239)	.008	1.535 (1.224-1.926)	<.0001
Hemoglobin	0.940 (0.866-1.020)	.140		
O_2_ saturation	0.908 (0.880-0.937)	<.001	0.989 (0.915-1.069)	.783
D-dimer	1.000 (1.000-1.000)	<.001	1.000 (1.000-1.000)	.006
Troponin	1.001 (0.998-1.003)	.534		
BNP	1.000 (1.000-1.000)	.183		

Abbreviations: BNP, B-type natriuretic peptide; DVT, deep venous thrombosis; CI, confidence interval; MPV, mean platelet volume; NLR, neutrophil-to-lymphocyte ratio; PAP, pulmonary artery pressure; PLR, platelet-to-lymphocyte ratio; PO, partial pressure of oxygen; PTE, pulmonary thromboembolism; sPESI, simplified Pulmonary Embolism Severity Index.

## Discussion

In our study, we investigated the association of the CHA2DS2-VASc score with the clinical subgroups of PTE with RVD and with in-hospital mortality. To the best of our knowledge, we have been the first to determine that the CHA2DS2-VASc score is an independent predictor of the RVD in acute PTE cases.

The acute PTE is an important clinical disease with high morbidity and mortality rates. It has been recognized that the presence of RVD is one of the most significant causes of death in deceased patients due to PTE.^15^ Hypoxia develops in acute PTE due to bronchoconstriction as the result of the neurohumoral changes caused by the reduction of pulmonary vascular bed and thrombus. Hypoxia causes a sudden increase in the pulmonary arterial pressure by creating vasoconstriction. On the other hand, the sudden rise in the pulmonary arterial pressure causes an increase in the right ventricle afterload, right ventricular dilatation, and eventually RVD. The significance of RVD in patients with acute PTE leads the investigators to study the predictors of this issue. A study conducted by Ates et al reported that PLR and NLR were found to be associated with the clinical severity of the patients with PTE.^16^ The role of the thrombocyte activity on the embolic events is well recognized. The increase in the thrombocyte activity in patients with acute PTE is associated with the increased severity of the clinical presentation of pulmonary embolism.^17^ The high-sensitivity cardiac troponin T and N-terminal pro B-type natriuretic peptide, which are the biochemical parameters performed at emergency departments, provide information on the RVD and can be used for this purpose.^18,19^ The importance of the RVD is more significant in patients presenting with the clinical signs and symptoms of submassive PTE rather than those presenting with massive PTE as detecting RVD in these patients or predicting the development of it during the follow-up will prompt the thrombolytic treatment option. In addition, the risk scores such as the sPESI are used to estimate the early mortality rate. The patients with sPESI ≥ 1 should be followed up closely and should be hospitalized for monitorization.^20^ Consistent with the literature, in our study too, the signs of right ventricular overload were observed to be more common in patients with sPESI > 1.

The effectiveness of this score in the treatment and follow-up processes in patients with atrial fibrillation has led to the hypothesis that this score can be used in other areas as well. Therefore, the score was matched against the outcomes of several diseases such as atrial fibrillation with pulmonary embolism, chronic obstructive pulmonary disease with or without AF, decreased left ventricular ejection fraction, and coronary artery disease.^12,21-25^ In this study, we firstly investigated the association of the CHA2DS2-VASc score with the RVD, clinical subgroups, and in-hospital mortality. In addition to the abovementioned studies, in this study, we found that the CHA2DS2-VASc score was significantly different among the clinical subgroups in patients with acute PTE and determined that the CHA2DS2-VASc score was an independent predictor of the RVD in these patients. However, it was found to be not associated with the in-hospital mortality in contrast to other studies.

The development of RVD in the settings of acute PTE has been related to several specific clinical and laboratory variables, such as diabetes, advanced age, and female gender.^26,27^ Risk scores incorporating these variables may be more accurate than those including either alone. Risk factors for RVD in patients with PTE are also included in the CHA2DS2-VASc score. The present study demonstrates that the CHA2DS2-VASc score is independently associated with the development of RVD in patients with acute PTE.

### Limitations of the Study

There are several limitations in our study. First, this study is retrospective, observational, and single-center study. Therefore, further studies are needed to draw definite conclusions. Second, we excluded patients on mechanical ventilation and hemodialysis; therefore, our results might not be applicable to those patients. Finally, our analysis involved a simple baseline determination at a single time point that may not reflect the patient status over long periods.

## Conclusion

Being independent of other factors, the CHA2DS2-VASc score is associated with the presence of RVD, which is an indicator of mortality. Further multicenter and large-scale studies are warranted for the score to be used in routine clinical practice.
